# Sodium-Glucose Cotransporter 2 Inhibitors Versus Dipeptidyl Peptidase-4 Inhibitors in Patients With Type 2 Diabetes Mellitus and Liver Cirrhosis: A Systematic Review and Meta-Analysis

**DOI:** 10.7759/cureus.109532

**Published:** 2026-05-24

**Authors:** Uday Shree Akkala Shetty, Ansharah-E-Zinnia Batool, Anastasia Postoev, Rahman Hameed Mohammed Abdul, Sonalben Chaudhary, Sidra Jabeen, Calvin R Wei, Adil Amin

**Affiliations:** 1 Internal Medicine, Southern Regional Medical Center, Riverdale, GEO; 2 Medicine, Rashid Latif Medical College, Lahore, PAK; 3 Internal Medicine, Caribbean Medical University, Willemstad, CUW; 4 Gastroenterology and Hepatology, King's Mill Hospital, Sutton-In-Ashfield, GBR; 5 Internal Medicine, Zydus Sitapur Hospital, Sitapur, IND; 6 Medicine, Latin American School of Medicine (ELAM), Sancti Spiritus, CUB; 7 Research and Development, Shing Huei Group, Taipei, TWN; 8 Cardiology, Pakistan Navy Ship Shifa, Karachi, PAK

**Keywords:** dpp-4 inhibitors, hepatic decompensation, liver cirrhosis, meta-analysis, sglt2 inhibitors

## Abstract

Liver cirrhosis complicated by type 2 diabetes mellitus (T2DM) is associated with significantly increased risks of hepatic decompensation, infection, and mortality. The optimal antidiabetic strategy in this population remains poorly defined, with limited evidence directly comparing available agents. This meta-analysis compared the clinical effectiveness of sodium-glucose cotransporter-2 inhibitors (SGLT2i) versus dipeptidyl peptidase-4 inhibitors (DPP4i) in patients with established liver cirrhosis. A systematic search of MEDLINE, EMBASE, Cochrane CENTRAL, and Web of Science was conducted up to 15 April 2026. Four retrospective observational cohort studies enrolling 25,000 patients with established cirrhosis and T2DM were included. All-cause mortality showed a pooled risk ratio of 0.64 (95% CI: 0.34-1.23; I^2^ = 89.1%), hepatic decompensation RR 0.75 (95% CI: 0.49-1.16; I^2^ = 84.9%), ascites HR 0.85 (95% CI: 0.55-1.29; I^2^ = 0.0%), oesophageal variceal bleeding RR 0.88 (95% CI: 0.59-1.30; I^2^ = 12.8%), and hepatic encephalopathy RR 0.89 (95% CI: 0.03-23.05; I^2^ = 83.4%). None of the pooled estimates reached statistical significance, largely attributable to the small number of contributing studies and substantial heterogeneity driven by aetiological diversity across cohorts. These results suggest that SGLT2i may be preferable to DPP4i in patients with compensated cirrhosis requiring antidiabetic intensification, though prospective randomised trials are needed to confirm this conclusion.

## Introduction and background

Liver cirrhosis is the end-stage consequence of chronic hepatic injury and represents a global health burden affecting more than 100 million individuals worldwide [1]. The condition is characterised by progressive hepatic fibrosis, architectural distortion, and portal hypertension, ultimately resulting in life-threatening complications including ascites, spontaneous bacterial peritonitis (SBP), hepatic encephalopathy (HE), oesophageal variceal haemorrhage, and hepatocellular carcinoma (HCC) [2]. The prognosis of patients with cirrhosis deteriorates markedly once hepatic decompensation occurs, with median survival falling to less than two years [3].

Type 2 diabetes mellitus (T2DM) is highly prevalent among patients with cirrhosis, affecting approximately 20-30% of this population [4,5]. This association is bidirectional: insulin resistance and hyperglycaemia contribute to the progression of chronic liver disease, while cirrhosis itself causes hepatogenous diabetes through portosystemic shunting, reduced hepatic insulin extraction, and impaired glycogen synthesis [6]. The coexistence of T2DM and cirrhosis is associated with a significantly greater risk of decompensation, infection, and death compared with either condition alone [7].

Despite the clinical importance of this comorbidity, the management of T2DM in cirrhosis remains challenging and evidence-poor. Metformin, insulin, and certain incretin-based therapies are used, but the impaired hepatic function, risk of lactic acidosis, altered drug metabolism, and susceptibility to hypoglycaemia substantially restrict therapeutic options [8]. Consequently, clear guidance on safe and effective antidiabetic therapy in cirrhosis is lacking, and most major randomised trials have systematically excluded this population [9].

Sodium-glucose cotransporter 2 inhibitors (SGLT2i) - including empagliflozin, dapagliflozin, and canagliflozin - exert insulin-independent glucose lowering by inhibiting renal tubular glucose reabsorption [10]. Beyond glycaemic control, SGLT2i have demonstrated robust cardioprotective and nephroprotective effects in large cardiovascular outcome trials [11,12]. Their potential role in liver disease has attracted growing interest given their pleiotropic mechanisms: natriuresis and osmotic diuresis (relevant to ascites management), reduction of hepatic lipogenesis and inflammation, attenuation of portal hypertension through neurohumoral modulation, and suppression of sympathetic nervous system activation [13,14].

Several observational studies and small pilot trials have reported that SGLT2i may reduce ascites burden, improve natriuresis, lower liver stiffness, and decrease rates of hepatic decompensation in patients with cirrhosis [15,16]. However, safety concerns persist, particularly regarding the risk of euglycaemic diabetic ketoacidosis (eDKA), acute kidney injury (AKI), volume depletion, and genitourinary infections in patients with advanced liver disease and already compromised renal and circulatory function [17].

Dipeptidyl peptidase-4 inhibitors (DPP4i) - including sitagliptin, linagliptin, saxagliptin, alogliptin, and vildagliptin - enhance endogenous incretin activity, stimulating glucose-dependent insulin secretion and suppressing glucagon [18]. DPP4i are weight-neutral and carry a low intrinsic risk of hypoglycaemia, rendering them theoretically attractive for use in cirrhosis [19]. However, most agents in this class undergo significant hepatic metabolism, and their pharmacokinetics are substantially altered in advanced liver disease [20]. Moreover, DPP4i have been associated with potential adverse cardiac effects in some trials, and they do not appear to offer meaningful benefits for liver-specific outcomes [21].

Several recent observational cohort studies have directly compared SGLT2i versus DPP4i in patients with established liver cirrhosis, reporting outcomes including mortality, hepatic decompensation, ascites, variceal haemorrhage, and HE [22,23]. While individual studies suggest a clinical advantage for SGLT2i, the body of evidence is heterogeneous in study design, population, follow-up duration, and comparator definition, making synthesis essential to inform clinical practice.

To date, no systematic review with meta-analysis has specifically synthesised the comparative effectiveness of SGLT2i versus DPP4i for liver-related clinical outcomes in patients with established cirrhosis and T2DM. Given the rapidly expanding evidence base and the significant unmet clinical need, the primary objective of this systematic review and meta-analysis is to evaluate the comparative effectiveness and safety of SGLT2i versus DPP4i in adults with established liver cirrhosis and co-existing T2DM.

## Review

Methodology

This systematic review and meta-analysis were conducted and reported in accordance with the Preferred Reporting Items for Systematic Reviews and Meta-Analyses (PRISMA) 2020 statement [24].

Information Sources and Search Strategy

A comprehensive electronic search was conducted in the following databases: MEDLINE (via PubMed), EMBASE (via Ovid), Cochrane Central Register of Controlled Trials (CENTRAL via Cochrane Library), Web of Science Core Collection, and ClinicalTrials.gov. The search covered all records from database inception to 15 April 2026. No language restriction was applied; non-English studies with available abstracts were considered for inclusion. In the event that potentially eligible non-English language studies were identified, translation would have been performed using validated online translation tools such as Google Translate, with subsequent verification by a bilingual co-author where possible.

The search strategy was developed in collaboration with a medical librarian and combined Medical Subject Headings (MeSH) terms and free-text keywords for: (1) SGLT2i (e.g., "sodium-glucose cotransporter 2 inhibitor", "empagliflozin", "dapagliflozin", "canagliflozin"); (2) DPP-4i (e.g., "dipeptidyl-peptidase 4 inhibitor", "sitagliptin", "linagliptin", "saxagliptin"); and (3) liver cirrhosis (e.g., "liver cirrhosis", "hepatic cirrhosis", "portal hypertension", "hepatic decompensation").

Reference lists of all included studies and relevant systematic reviews identified during screening were manually searched for additional eligible studies. Authors of relevant studies were contacted to identify unpublished data where appropriate.

Study Selection

All retrieved records were imported into EndNote (Clarivate, London, UK). Duplicate records were identified and removed. Two independent reviewers (blinded to each other's decisions) screened titles and abstracts against the eligibility criteria. Full-text articles were obtained for all potentially eligible studies. Two reviewers independently assessed full texts for eligibility, and any discrepancies were resolved through discussion and, when necessary, arbitration by a third reviewer. The study selection process was documented in a PRISMA 2020 flow diagram.

Eligibility Criteria

Eligible studies enrolled adults (≥18 years) with established liver cirrhosis - confirmed by histology, clinical criteria, validated non-invasive scores, or imaging - and co-existing T2DM. No restriction was applied by cirrhosis aetiology (viral hepatitis B/C, alcohol-related, metabolic dysfunction-associated steatotic liver disease/metabolic dysfunction-associated steatohepatitis (MASLD/MASH), cryptogenic, or other) or by Child-Pugh or Model for End-Stage Liver Disease (MELD) score. Studies enrolling patients with pre-cirrhotic liver disease, or those in which cirrhosis was an endpoint rather than an entry criterion, were excluded. The intervention was any SGLT2i compared with any DPP-4i as the active comparator. Pre-specified primary outcomes were all-cause mortality, composite hepatic decompensation, and incident or worsening ascites. Secondary outcomes included HE and oesophageal variceal haemorrhage. RCTs, quasi-randomised trials, prospective cohort studies, and retrospective cohort studies with an active comparator design were eligible; case reports, narrative reviews, and conference abstracts without extractable data were excluded.

Data Extraction

Data were extracted using a data extraction form developed in Microsoft Excel (Microsoft Corp., Redmond, WA, USA). Data were extracted by two authors independently. Any disagreement between authors was resolved through discussion. Data extracted from included studies comprised: author name, year, region, study design, groups, sample size, follow-up, mean age, gender (number of males), and outcome data.

Risk of Bias Assessment

Risk of bias in included observational studies was assessed using the Newcastle-Ottawa Scale (NOS), which evaluates three domains: selection of study groups, comparability of groups, and ascertainment of outcome [25]. Studies scoring ≥7 out of 9 stars were considered to have a low risk of bias; those scoring 5-6 stars were considered moderate; and those scoring ≤4 stars were considered high risk. For RCTs (if identified), the Cochrane Risk of Bias 2 (RoB 2) tool was applied, assessing five domains: randomisation process, deviations from intended interventions, missing outcome data, measurement of the outcome, and selection of reported results [26].

Data Analysis

All statistical analyses were performed using R (version 4.4.0; R Foundation for Statistical Computing, Vienna, Austria) with the meta and metafor packages. A two-sided p-value of <0.05 was considered statistically significant for all analyses. For categorical outcomes, the risk ratio (RR) was reported along with the 95% confidence interval (CI). SGLT2i served as the intervention and DPP4i as the reference group in all comparisons; estimates below 1.0 therefore indicate a benefit associated with SGLT2i use. Pooled effect estimates were calculated using the DerSimonian-Laird random-effects model, which was selected a priori given the anticipated clinical and methodological diversity across included studies. The Hartung-Knapp-Sidik-Jonkman (HKSJ) correction was applied to the CIs of all pooled estimates to account for uncertainty in the estimation of between-study variance, particularly when the number of included studies was small (k ≤ 5). Between-study statistical heterogeneity was quantified using the I^2^ statistic, the Cochran Q test p-value, and the τ^2^ (tau-squared) estimate of between-study variance. I^2^ values were interpreted according to conventional thresholds: 0-25% as low, 26-50% as moderate, 51-75% as substantial, and >75% as considerable heterogeneity.

Results

**Figure 1:**
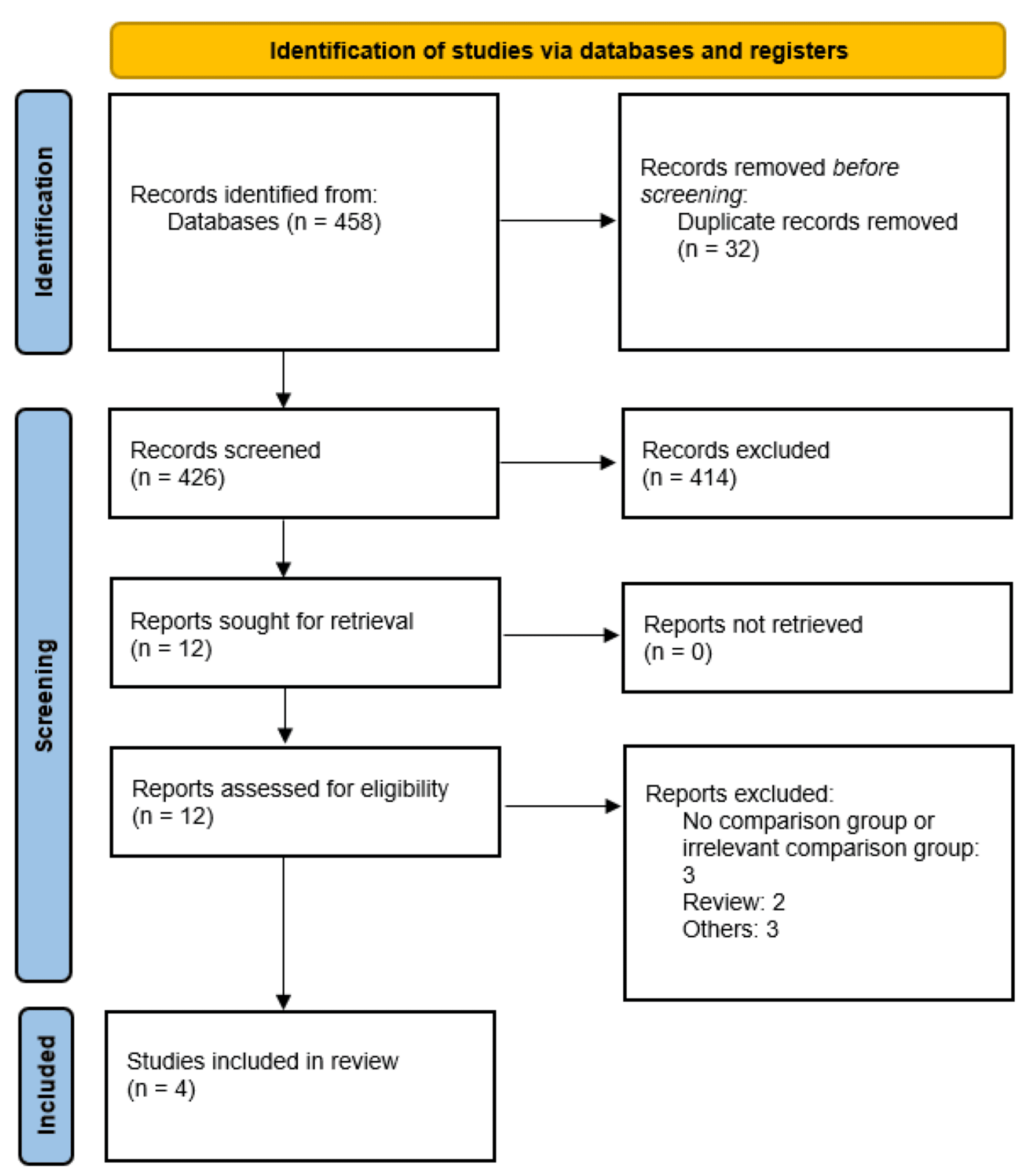
PRISMA flowchart

Through searches of multiple online databases, a total of 458 studies were identified. After removing duplicate records, 426 studies remained for initial screening based on their titles and abstracts. Subsequently, the full texts of 12 studies were retrieved and assessed in detail according to the predefined eligibility criteria. Ultimately, four studies met the inclusion criteria and were included in the meta-analysis. The study selection process is illustrated in Figure 1, which presents the PRISMA flow diagram of study identification and inclusion. Table 1 presents characteristics of included studies. Table 2 presents a quality assessment of included studies.

**Table 1:** Characteristics of included studies DPP4i: dipeptidyl peptidase-4 inhibitors; EHR: electronic health records; IBM: International Business Machines (MarketScan database provider); N: total sample size; n: number of participants in subgroup; NHIRD: National Health Insurance Research Database; SGLT2i: sodium-glucose cotransporter-2 inhibitors; TriNetX: TriNetX Global Collaborative Network; USA: United States of America; VA: Veterans Affairs; VOCAL: Veterans Outcomes and Costs Associated with Liver Disease; yrs: years

Author (Year)	Region	Study Design	Data Source	Intervention (SGLT2i)	Comparator (DPP4i)	Total N	Follow-Up	Mean Age (Years)	Male (n)
Chou et al., 2026 [27]	Multi-national (TriNetX Global Network; predominantly North America and Europe)	Retrospective Cohort	TriNetX Global Collaborative Network (>120 healthcare organisations worldwide; 2016-2024)	SGLT2i (any) n = 5,398	DPP4i (any) n = 5,398	N= 10796	49.8 months	SGLT2i: 63.9 DPP4i: 64.0	SGLT2i: 2,880 DPP4i: 2,893
Chung et al., 2026 [28]	Taiwan	Retrospective Cohort	Taiwan National Health Insurance Research Database (NHIRD; 23 million citizens; May 2016-December 2023)	SGLT2i (dapagliflozin, empagliflozin, canagliflozin) n = 9,689	DPP4i (alogliptin, linagliptin, sitagliptin, saxagliptin, vildagliptin) n = 14,570	N = 24,259	2.3 years	SGLT2i: 62.25 DPP4i: 66.29	SGLT2i: 6,980 DPP4i: 9,050
Saffo et al., 2021 [22]	USA	Retrospective Cohort	VA VOCAL Database (Electronic Health Records, 130,000 veterans; 2008-2020)	SGLT2i (empagliflozin) n = 423	DPP4i (alogliptin, linagliptin, saxagliptin, sitagliptin) n = 423	N = 846	36 Months	SGLT2i: 67 yrs DPP4i: 66 yrs	SGLT2i: 418 DPP4i: 417
Simon et al., 2022 [29]	USA	Retrospective Cohort	IBM MarketScan (Commercial claims database; 2013-2020)	SGLT2i (canagliflozin, dapagliflozin, empagliflozin, ertugliflozin) n = 845	DPP4i (alogliptin, linagliptin, saxagliptin, sitagliptin) n = 845	N= 1690	12 Months	SGLT2i: 58.1 yrs DPP4i: 58.0 yrs	SGLT2i: 498 DPP4i: 502

**Table 2:** Quality assessment using Newcastle-Ottawa Scale

Author (Year)	Selection	Comparison	Outcome	Overall
Chou et al., 2026 [27]	4	2	3	Good
Chung et al., 2026 [28]	4	2	2	Good
Saffo et al., 2021 [22]	4	2	2	Good
Simon et al., 2022 [29]	4	1	1	Moderate

Meta-Analysis of Outcomes

**Figure 2:**
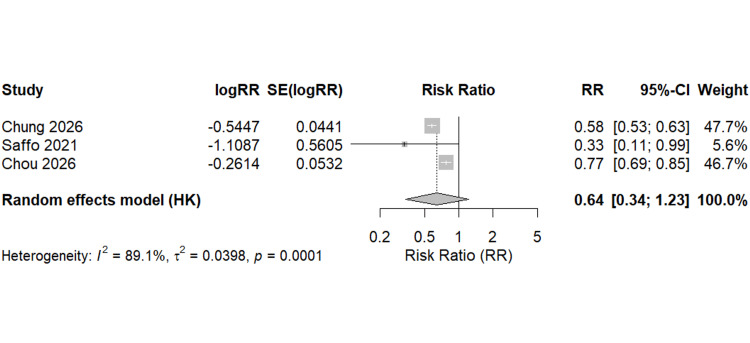
Comparison of mortality between SGLT2i and DPP-4 inhibitors SGLT2i: sodium-glucose cotransporter-2 inhibitors; DPP4i: dipeptidyl peptidase-4 inhibitors; HK: Hartung-Knapp References [22,27,28]

All-cause mortality: Three studies reported data on all-cause mortality. The pooled risk ratio using a random-effects model was RR 0.64 (95% CI: 0.34-1.23), indicating a trend towards reduced mortality with SGLT2i compared with DPP4i, although this did not reach statistical significance. Substantial heterogeneity was observed (I^2^ = 89.1%, τ^2^ = 0.0398, p = 0.0001), reflecting meaningful differences in study populations, follow-up duration, and case-mix severity as shown in Figure 2. Individual study estimates ranged from RR 0.33 (95% CI: 0.11-0.99) in Saffo 2021 to RR 0.77 (95% CI: 0.69-0.85) in Chou 2026, with Chung 2026 reporting an estimate of RR 0.58 (95% CI: 0.53-0.63). Despite consistent directional trends favouring SGLT2i, the wide CI of the pooled estimate precludes a definitive conclusion.

Hepatic Decompensation Events

**Figure 3:**
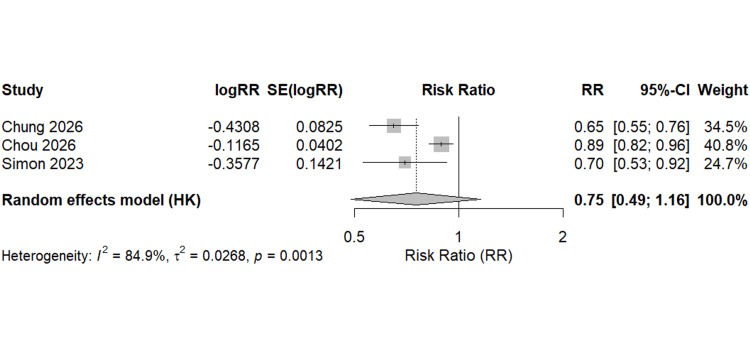
Comparison of decomposition events between SGLT2i and DPP-4 inhibitors SGLT2i: sodium-glucose cotransporter-2 inhibitors; DPP4i: dipeptidyl peptidase-4 inhibitors; HK: Hartung-Knapp References [27-29]

Three studies contributed data on composite hepatic decompensation events. The pooled risk ratio was RR 0.75 (95% CI: 0.49-1.16), trending in favour of SGLT2i without achieving statistical significance. Considerable heterogeneity was present (I^2^ = 84.9%, τ^2^ = 0.0268, p = 0.0013). Individual estimates ranged from RR 0.65 (95% CI: 0.55-0.76) in Chung 2026 to RR 0.89 (95% CI: 0.82-0.96) in Chou 2026, as shown in Figure 3. All three studies independently demonstrated a reduction in decompensation events with SGLT2i, suggesting a clinically meaningful signal attenuated in the pooled estimate due to between-study heterogeneity in cirrhosis severity, baseline decompensation rates, and follow-up periods.

Other Outcomes

Three additional outcomes were analysed: ascites, oesophageal variceal bleeding, and HE. For ascites, pooled analysis yielded a pooled RR of 0.85 (95% CI: 0.55-1.29) with negligible heterogeneity (I^2^ = 0.0%), indicating a consistent but non-significant trend favouring SGLT2i. For oesophageal variceal bleeding, three studies produced a pooled RR of 0.88 (95% CI: 0.59-1.30) with low heterogeneity (I^2^ = 12.8%). For HE, only two studies contributed data, and the pooled RR of 0.89 (95% CI: 0.03-23.05) was rendered uninterpretable by extreme heterogeneity (I^2^ = 83.4%) and divergent individual estimates. A summary of pooled estimates is provided in Table 3.

**Table 3:** Summary of pooled estimates for other outcomes (SGLT2i vs. DPP4i) All estimates from the random-effects model with the Hartung-Knapp (HK) correction. SGLT2i: sodium-glucose cotransporter-2 inhibitors; DPP4i: dipeptidyl peptidase-4 inhibitors; RR: risk ratio; CI: confidence interval; I^2^: between-study heterogeneity statistic

Outcome	References	Pooled RR	95% CI	I^2^ (%)
Ascites	[22-27]	0.85	0.55-1.29	0.00
Oesophageal variceal bleeding	[27-29]	0.88	0.59-1.30	12.80
Hepatic encephalopathy	[27,29]	0.89	0.03-23.05	83.40

Discussion

To our knowledge, this is the first systematic review and meta-analysis to specifically compare SGLT2i against DPP-4i across multiple liver-related clinical outcomes in patients with established cirrhosis and T2DM. Across four retrospective cohort studies enrolling over 25,000 patients, our pooled analyses consistently pointed in the same direction: SGLT2i were associated with numerically lower risks of death, hepatic decompensation, ascites, oesophageal variceal bleeding, and HE compared with DPP4i. Importantly, however, none of the pooled estimates attained formal statistical significance, and substantial between-study heterogeneity was observed for mortality (I^2^ = 89.1%) and decompensation (I^2^ = 84.9%). These findings must therefore be interpreted as hypothesis-generating rather than conclusive, underscoring an urgent need for prospective evidence.

The consistent directional signal favouring SGLT2i across all five outcomes is biologically plausible and aligns with several mechanistic pathways relevant to cirrhosis. SGLT2 inhibition induces natriuresis and osmotic diuresis independent of aldosterone, offering a renal sodium-handling advantage that complements but does not replicate conventional diuretic therapy [10]. In the context of cirrhosis, where renal sodium retention driven by portal hypertension and neurohormonal activation underpins ascites formation, this mechanism is particularly pertinent [30]. Furthermore, SGLT2i suppress sympathetic nervous system activity and the renin-angiotensin-aldosterone system - both of which are chronically over-activated in decompensated cirrhosis - potentially attenuating haemodynamic deterioration and reducing the threshold for decompensation [31]. These properties stand in contrast to DPP4i, which act primarily through incretin enhancement and confer no meaningful haemodynamic or natriuretic benefit in this population [18].

The broader literature supports these findings. Mantovani et al., in a meta-analysis of eight observational cohort studies in patients with T2DM, reported that SGLT2i use was associated with a 17% reduction in major adverse liver-related outcomes, including a significant reduction in incident cirrhosis and HCC, compared with non-use [32]. Critically, however, that analysis was not restricted to patients with established cirrhosis and did not employ DPP4i as the primary active comparator - two limitations that our meta-analysis directly addresses. The Kim et al. TriNetX study of 6,449 patients with MASH cirrhosis and T2DM similarly demonstrated that SGLT2i were associated with significantly lower risks of all-cause mortality (HR 0.58), hepatic decompensation (HR 0.85), and major adverse liver outcomes (HR 0.88) compared with other glucose-lowering drugs [33]. Simon et al. further demonstrated that SGLT2i added to standard diuretics significantly reduced composite serious liver events - including ascites, variceal development, and all-cause mortality - in cirrhotic patients already receiving furosemide and spironolactone [29]. Taken together, these data from diverse populations and comparator groups converge on a consistent hepatoprotective signal that our meta-analysis quantifies and contextualises.

The mortality signal in our analysis (RR 0.64) is most robustly anchored by the Chung et al. Taiwan NHIRD cohort of 24,259 patients, which reported a 42% reduction in all-cause mortality (aHR 0.58, 95% CI: 0.53-0.63) with SGLT2i versus DPP4i, with protective associations that remained consistent across viral, alcohol-related, and MASLD-related cirrhosis subgroups [28]. The notably wide pooled CI is a direct methodological consequence of the Saffo 2021 cohort contributing only 5.6% of pooled weight - attributable to its small matched sample (n = 846) arising from the stringent requirement for concurrent metformin use [22]. This reflects a genuine methodological challenge of pooling studies with markedly different sample sizes and precision, and should not be interpreted as evidence of effect inconsistency, given that all three individual mortality estimates fall below 1.0.

For hepatic decompensation, the pooled RR of 0.75 is supported by consistent individual signals. Chung et al. reported a 35% reduction in composite decompensation events (aHR 0.65, 95% CI: 0.57-0.74), encompassing specific reductions in ascites, peritonitis, and oesophageal variceal bleeding [28], while Simon et al. independently demonstrated a significant reduction in the composite of serious liver events across a multi-institutional TriNetX cohort [29]. The substantial heterogeneity (I^2^ = 84.9%) reflects genuine differences in how decompensation was defined, the prevalence of pre-existing ascites at baseline, and the proportion of patients with Child-Pugh B/C disease across cohorts, rather than true biological inconsistency. Crucially, all three contributing studies demonstrated point estimates below 1.0, which strengthens biological plausibility even without a statistically significant pooled estimate. This mirrors the early meta-analytic pattern seen with SGLT2i in heart failure, where directional observational signals preceded formal confirmatory trial evidence.

The pooled ascites estimate (HR 0.85, I^2^ = 0.0%) is particularly noteworthy. The complete absence of heterogeneity across two aetiologically distinct populations - a US veteran cohort dominated by alcohol-related cirrhosis and a Taiwanese cohort with predominantly viral and MASLD-related disease - indicates that the directional effect on ascites is stable and population-independent. This is mechanistically corroborated by interventional evidence: Kalambokis et al. demonstrated in a pilot trial that empagliflozin improved natriuresis, reduced ascites burden, and attenuated circulatory dysfunction in refractory ascites [15], and Singh et al. reported that dapagliflozin significantly reduced paracentesis frequency and body weight in recurrent ascites [16]. The oesophageal variceal bleeding estimate (RR 0.88, I^2^ = 12.8%) similarly shows low heterogeneity across three geographically diverse cohorts, suggesting a reproducible directional effect possibly mediated through attenuation of portal hypertension-driven haemodynamic changes rather than any population-specific drug effect. In contrast, the HE result (RR 0.89, I^2^ = 83.4%) is rendered uninterpretable by the opposing estimates of Chou 2026 (RR 1.11) and Simon et al (RR 0.66), likely reflecting differences in baseline ammonia burden, concurrent lactulose and rifaximin use, and diagnostic ascertainment methodology - underscoring a critical unresolved evidence gap. Additionally, the opposing estimates for HE between Chou 2026 and Simon 2022 most plausibly reflect differences in background rifaximin and lactulose use, unmeasured ammonia burden, and the limited biological rationale for SGLT2i to directly modulate ammonia metabolism or the gut-liver-brain axis, rather than a true pharmacological inconsistency. This outcome should be considered an unresolved evidence gap requiring prospective standardised evaluation.

The high heterogeneity observed for mortality (I^2^ = 89.1%) and hepatic decompensation (I^2^ = 84.9%) is attributable to aetiological diversity across cohorts - predominantly alcohol-related cirrhosis in Saffo 2021, viral hepatitis and MASLD in the Taiwanese cohorts, and a mixed multinational population in Chou 2026 - and to differences in cirrhosis severity, follow-up duration, and outcome definitions. Formal subgroup meta-analyses stratified by cirrhosis aetiology or Child-Pugh/MELD severity were not feasible given the absence of extractable stratum-specific effect estimates across studies and the small number of contributing studies per outcome. Individual patient data meta-analysis, which would enable such analyses, is identified as a priority for future research.

Clinical Implications

The uniform directionality across five independent outcomes, combined with a biologically coherent mechanism and an active comparator design that substantially reduces confounding by indication, provides sufficient grounds to favour SGLT2i over DPP4i when antidiabetic intensification is required in compensated cirrhosis. This is particularly relevant given the paucity of safe options: metformin is contraindicated in decompensated disease, sulfonylureas carry hypoglycaemia risk, and insulin is metabolically unpredictable in the context of impaired hepatic glucose regulation [8,9]. SGLT2i should be avoided in Child-Pugh C disease or active decompensation, with close monitoring for euglycaemic ketoacidosis, AKI, and volume depletion in all cirrhotic patients.

Limitations

All included studies were retrospective observational cohorts, precluding causal inference. Residual confounding by cirrhosis severity persists despite propensity score adjustment, as SGLT2i may have been preferentially prescribed to less advanced patients. The small number of studies per outcome (two to three) limited statistical power and precluded publication bias assessment. Substantial heterogeneity for mortality and decompensation was driven by aetiological diversity - alcohol-related in Saffo 2021, viral hepatitis-predominant in the Taiwanese cohorts, and MASLD in Abu-Hammour 2025. Cirrhosis severity by Child-Pugh or MELD was inconsistently reported, preventing disease-stage subgroup analyses.

Future Directions

Prospective randomised trials comparing SGLT2i with DPP4i in established cirrhosis, stratified by Child-Pugh class and aetiology, are a research priority. Standardised, graded definitions for HE are essential given the discordance between the contributing studies. Trials should include variceal events, ascites-free survival, and HCC as pre-specified long-term outcomes. Individual patient data meta-analysis would enable subgroup analyses by cirrhosis severity and specific SGLT2i agent that the current study-level analysis could not perform.

## Conclusions

This meta-analysis synthesised data from four retrospective cohort studies encompassing over 25,000 patients with T2DM and established liver cirrhosis, providing the first pooled comparison of SGLT2i versus DPP4i across five liver-related outcomes. Although none of the pooled estimates achieved statistical significance, a consistent directional trend favouring SGLT2i was observed across all outcomes, including all-cause mortality, hepatic decompensation, ascites, oesophageal variceal bleeding, and HE. The biological plausibility of these findings, supported by the natriuretic, neurohumoral, and antifibrotic mechanisms of SGLT2i, alongside the use of an active comparator design, strengthens confidence in the observed signal. These findings support preferential use of SGLT2i over DPP4i in patients with compensated cirrhosis requiring antidiabetic intensification. Prospective randomised trials are warranted to confirm this observation.
